# Supplementary material for: Early-stage health technology assessment of fractional flow reserve coronary computed tomography versus standard diagnostics in patients with stable chest pain in The Netherlands
Source: PLoS One. 2024 Jun 13;19(6):e0305189. doi: 10.1371/journal.pone.0305189 (PMC11175410; doi:10.1371/journal.pone.0305189)
Supplement: S1 Table — Abbreviations: ICA: invasive coronary angiography; OR: operating room; FFR: fractional flow reserve; MRI: magnetic resonance imaging; IV: intravenous; CCTA: coronary computed tomography angiography; FFRct: fractional flow reserve from coronary computed tomography. (DOCX) [file pone.0305189.s001.docx]

**S1 Table.** **Dutch unit costs, resource quantities, and total costs of the diagnostic procedures**

|  | **Unit cost (€)** | **Resource quantity** | **Total costs (€) per procedure** | **Source** |
| --- | --- | --- | --- | --- |
| **ICA** |  |  |  |  |
| Disposables: |  |  |  |  |
| Guiding catheter | 133.83 | 1 | 133.83 | [1] |
| Guidewire | 157.04 | 1 | 157.04 | [1] |
| Contrast agent | 1.37 | 1 | 1.37 | [1] |
| Sterile clothing | 138.90 | 3 | 416.71 |  |
| Hygiene apron | 138.05 |  |  | [2] |
| Gloves | 0.24 |  |  | [2] |
| Mask | 0.22 |  |  | [3] |
| Hair cover | 0.40 |  |  | [3] |
| Plastic sheet | 4.53 | 2 | 9.06 | [3] |
| Needle | 0.05 | 1 | 0.05 | [2] |
| Anesthetic shot | 5.23 | 1 | 5.23 | [4] |
| Disinfect | 0.29 | 1 | 0.29 | [4] |
| Protection foil | 2.58 | 3 | 7.74 | [3] |
| Hospital costs: |  |  |  |  |
| Hospital day | 542.64 | 1 | 542.64 | [5] |
| Outpatient visit | 103.74 | 1 | 103.74 | [5] |
| Medical specialist (per hour); Mean | 141.31 | 1 | 141.31 |  |
| Medical specialist; non-academic hospital (per hour) | 143.16 |  |  | [5] |
| Medical specialist; academic hospital (per hour) | 139.46 |  |  | [5] |
| Hybrid OR per minute | 21.86 | 30 | 655.94 | [6] |
|  |  |  |  |  |
| **FFR(ICA)** |  |  |  |  |
| Pressure wire | 754.02 | 1 | 754.02 | [7] |
| Hybrid OR per minute | 21.86 | 3 | 65.58 | [6] |
|  |  |  |  |  |
| ***Total costs ICA visual*** |  |  | ***2,174.95*** |  |
| ***Total costs ICA FFR*** |  |  | ***2,994.55*** |  |
|  |  |  |  |  |
| **MRI** |  |  |  |  |
| Disposables: |  |  |  |  |
| Contrast agent | 1.37 | 1 | 1.37 | [8] |
| IV needle | 1.65 | 1 | 1.65 | [2] |
| Needle | 0.05 | 1 | 0.05 | [2] |
| Blood tube | 0.30 | 1 | 0.30 | [2] |
| Hospital costs: |  |  |  |  |
| MRI scan | 261.06 | 1 | 261.06 | [5] |
| Adenosine | 3.71 | 1 | 3.71 | [4] |
| Medical specialist (per hour); Mean | 141.31 | 1 | 141.31 | [5] |
| Medical specialist; non-academic hospital (per hour) | 143.16 |  |  |  |
| Medical specialist; academic hospital (per hour) | 139.46 |  |  | [5] |
| Outpatient visit | 103.74 | 2 | 207.48 | [5] |
|  |  |  |  |  |
| ***Total costs MRI*** |  |  | ***616.93*** |  |
|  |  |  |  |  |
| **CCTA** |  |  |  |  |
| Disposables: |  |  |  |  |
| Contrast agent | 1.37 | 1 | 1.37 | [8] |
| IV needle | 1.65 | 1 | 1.65 | [2] |
| Needle | 0.05 | 1 | 0.05 | [2] |
| Blood tube | 0.30 | 1 | 0.30 | [2] |
| Hospital costs: |  |  |  |  |
| CT scan | 165.30 | 1 | 165.30 | [5] |
| Metoprolol | 1.76 | 1 | 1.76 | [4] |
| Outpatient visit | 103.74 | 2 | 207.48 | [5] |
| Medical specialist (per hour); Mean | 141.31 | 1 | 141.31 |  |
| Medical specialist; non-academic hospital (per hour) | 143.16 |  |  | [5] |
| Medical specialist; academic hospital (per hour) | 139.46 |  |  | [5] |
|  |  |  |  |  |
| **FFRct** |  |  |  |  |
| FFR analysis | 700.00 | 1 | 700.00 |  |
|  |  |  |  |  |
| ***Total costs CCTA*** |  |  | ***519.22*** |  |
| ***Total costs FFRct*** |  |  | ***1,219.22*** |  |

Abbreviations: ICA: invasive coronary angiography; OR: operating room; FFR: fractional flow reserve; MRI: magnetic resonance imaging; IV: intravenous; CCTA: coronary computed tomography angiography; FFRct: fractional flow reserve from coronary computed tomography

**References**

1. van Hout, B.A., et al., *One year cost effectiveness of sirolimus eluting stents compared with bare metal stents in the treatment of single native de novo coronary lesions: an analysis from the RAVEL trial.* Heart, 2005. **91**(4): p. 507-12.

2. Medeco, P.M. *Medical disposables and equipment for general practitioners, midwives, and physiotherapists*. March 22, 2023]; Available from: <https://pluspunt.mediqmedeco.nl>

3. Klinimed. [cited March 22, 2023; Available from: <https://www.klinimed.nl>

4. Dutch Pharmacotherapeutic Compass, N.H.C.I.Z.N. March 22, 2023; Available from: <https://www.farmacotherapeutischkompas.nl>

5. Hakkaart-van Roijen, L., et al., *Kostenhandleiding.* Methodologie van kostenonderzoek en referentieprijzen voor economische evaluaties in de gezondheidszorg In opdracht van Zorginstituut Nederland Geactualiseerde versie, 2015: p. 12-64.

6. Patel, S., et al., *Understanding the Costs of Surgery: A Bottom-Up Cost Analysis of Both a Hybrid Operating Room and Conventional Operating Room.* Int J Health Policy Manag, 2022. **11**(3): p. 299-307.

7. Fearon, W.F., et al., *Economic evaluation of fractional flow reserve-guided percutaneous coronary intervention in patients with multivessel disease.* Circulation, 2010. **122**(24): p. 2545-50.

8. Hlatky, M.A., et al., *Projected costs and consequences of computed tomography-determined fractional flow reserve.* Clin Cardiol, 2013. **36**(12): p. 743-8.
