# Supplementary material for: Early-stage health technology assessment of fractional flow reserve coronary computed tomography versus standard diagnostics in patients with stable chest pain in The Netherlands
Source: PLoS One. 2024 Jun 13;19(6):e0305189. doi: 10.1371/journal.pone.0305189 (PMC11175410; doi:10.1371/journal.pone.0305189)
Supplement: S2 Table — *The steps of administering medications are described in the guidelines for (maintenance) treatment for patients with stable angina pectoris by the National Health Care Institute in The Netherlands [13]. (DOCX) [file pone.0305189.s002.docx]

**S2 Table.** **Mean costs of medications for patients with stable angina pectoris.**

|  | **Step*** | **Description** | **Costs (€)**  **per dose** | **Mean costs (€) per patient per year** |
| --- | --- | --- | --- | --- |
| **Medications after non-invasive testing** | **1** | Non-medical treatment |  |  |
|  | **2** | Acetylsalicylic acid | 0.04 | 21.90 |
|  | OR | Clopidogrel | 0.08 |  |
|  | **3** | Bisoprolol | 0.05 | 34.68 |
|  | OR | Metoprolol | 0.15 |  |
|  | OR | Amlodipine | 0.04 |  |
|  | OR | Felodipine | 0.14 |  |
|  | ***Mean cost medications after non-invasive testing*** | | | ***28.29*** |
| **Medications after invasive testing** | **4** | Discuss therapy loyalty |  |  |
|  | **5a** | Bisoprolol | 0.05 | 69.35 |
|  | OR | Metoprolol | 0.15 |  |
|  |  | AND |  |  |
|  |  | Amlodipine | 0.04 |  |
|  | OR | Felodipine | 0.14 |  |
|  | **5b** | AND |  | 156.95 |
|  |  | Isosorbide mononitrate | 0.13 |  |
|  | OR | Isosorbide dinitrate | 0.54 |  |
|  | **6** | Bisoprolol | 0.05 | 191.63 |
|  | OR | Metoprolol | 0.15 |  |
|  |  | AND |  |  |
|  |  | Amlodipine | 0.04 |  |
|  | OR | Felodipine | 0.14 |  |
|  |  | AND |  |  |
|  |  | Isosorbide mononitrate | 0.13 |  |
|  | OR | Isosorbide dinitrate | 0.54 |  |
|  | ***Mean cost medications after invasive testing*** | | | ***139.31*** |

*The steps of administering medications are described in the guidelines for (maintenance) treatment for patients with stable angina pectoris by the National Health Care Institute in The Netherlands [1].

**References**

1. Nederland Z. Onderhoudsbehandeling van stabiele angina pectoris [20-01-2022]. Available from: <https://www.farmacotherapeutischkompas.nl/bladeren/indicatieteksten/coronairlijden?anchor=coronairlijden_onderhoudsbehandeling_van_stabiele_angina_pectoris>.
