# Supplementary material for: Early-stage health technology assessment of fractional flow reserve coronary computed tomography versus standard diagnostics in patients with stable chest pain in The Netherlands
Source: PLoS One. 2024 Jun 13;19(6):e0305189. doi: 10.1371/journal.pone.0305189 (PMC11175410; doi:10.1371/journal.pone.0305189)
Supplement: S1 File — (DOCX) [file pone.0305189.s003.docx]

**S3 File. Calculations of sensitivity and specificity per diagnostic tool based on the generic inverse variance weighted average [13].**

G$eneric inverse-variance wighted average=\frac{\sum Y_{i}(\frac{1}{{SE}_{i}^{2}})}{\sum(\frac{1}{{SE}_{i}^{2}})}$

**Table 1. Sensitivity of CCTA**

| *Sn* | *95% CI* | *n* | *SE* | *Source* |
| --- | --- | --- | --- | --- |
| 93.8 | 79.9-98.3 | 61 | 4.69 | [1] |
| 89 | 81-95 | 157 | 3.57 | [2] |
| 90 | 86-93 | 694 | 1.79 | [3] |
|  |  |  |  |  |
| Weighted average: | | **90.22** |  |  |

**Table 2. Specificity of CCTA**

| *Sp* | *95% CI* | *n* | *SE* | *Source* |
| --- | --- | --- | --- | --- |
| 72.4 | 54.3-85.3 | 61 | 7.91 | [1] |
| 63 | 52-73 | 157 | 5.36 | [2] |
| 71 | 65-75 | 609 | 2.55 | [3] |
|  |  |  |  |  |
| Weighted average: | | **45.05** |  |  |

**Table 3. Sensitivity of FFRct**

| *Sn* | *95% CI* | *n* | *SE* | *Source* |
| --- | --- | --- | --- | --- |
| 93,8 | 79.9-98.3 | 61 | 4.69 | [1] |
| 97 | 91-99 | 157 | 2.04 | [2] |
| 90 | 85-93 | 609 | 2.04 | [3] |
|  |  |  |  |  |
| Weighted average: | | **93.53** |  |  |

**Table 4. Specificity of FFRct**

| *Sp* | *95% CI* | *n* | *SE* | *Source* |
| --- | --- | --- | --- | --- |
| 72.4 | 54.3-85.3 | 61 | 7.91 | [1] |
| 63 | 52-73 | 157 | 5.36 | [2] |
| 71 | 65-75 | 609 | 2.55 | [3] |
|  |  |  |  |  |
| Weighted average: | | **69.75** |  |  |

Sensitivity and specificity values of MRI and ICA were based on one source [3] and therefore not calculated with the aforementioned formula.

**References**

1. Peper, J., et al., *Diagnostic accuracy of on-site coronary computed tomography-derived fractional flow reserve in the diagnosis of stable coronary artery disease.* Neth Heart J, 2022. **30**(3): p. 160-171.

2. Driessen, R.S., et al., *Comparison of Coronary Computed Tomography Angiography, Fractional Flow Reserve, and Perfusion Imaging for Ischemia Diagnosis.* J Am Coll Cardiol, 2019. **73**(2): p. 161-173.

3. Danad, I., et al., *Diagnostic performance of cardiac imaging methods to diagnose ischaemia-causing coronary artery disease when directly compared with fractional flow reserve as a reference standard: a meta-analysis.* Eur Heart J, 2017. **38**(13): p. 991-998.
