# Supplementary material for: Early-stage health technology assessment of fractional flow reserve coronary computed tomography versus standard diagnostics in patients with stable chest pain in The Netherlands
Source: PLoS One. 2024 Jun 13;19(6):e0305189. doi: 10.1371/journal.pone.0305189 (PMC11175410; doi:10.1371/journal.pone.0305189)
Supplement: Attachment — Submitted filename: Response to reviewers.docx [file pone.0305189.s004.docx]

When submitting your revision, we need you to address these additional requirements.

1. Please ensure that your manuscript meets PLOS ONE's style requirements, including those for file naming. The PLOS ONE style templates can be found at

https://journals.plos.org/plosone/s/file?id=wjVg/PLOSOne_formatting_sample_main_body.pdf and 
https://journals.plos.org/plosone/s/file?id=ba62/PLOSOne_formatting_sample_title_authors_affiliations.pdf

2. Please note that PLOS ONE has specific guidelines on code sharing for submissions in which author-generated code underpins the findings in the manuscript. In these cases, all author-generated code must be made available without restrictions upon publication of the work. Please review our guidelines at https://journals.plos.org/plosone/s/materials-and-software-sharing#loc-sharing-code and ensure that your code is shared in a way that follows best practice and facilitates reproducibility and reuse.

No specific code has been used to perform our analyses. The search term used in PubMed for our literature review is added to the methods section.

Please see page 6, line 99-103:

*To collect data on costs and effects a literature search was performed in Pubmed using the following search term: (((coronary artery disease[MeSH Terms]) AND (cardiac diagnostic method[MeSH Terms])) AND (("2015/01/01"[Date - Completion] : "3000"[Date - Completion]))) AND ((coronary angiography[MeSH Terms]) OR (fractional flow reserve, myocardial[MeSH Terms]) OR (computed tomography[MeSH Terms])).*

3. Please be informed that funding information should not appear in the Acknowledgments section or other areas of your manuscript. We will only publish funding information present in the Funding Statement section of the online submission form. Please remove any funding-related text from the manuscript.

Funding statement removed from pages 17-18, lines 340-344.

4. We note that your Data Availability Statement is currently as follows: "All relevant data are within the manuscript and its Supporting Information files."

Please confirm at this time whether or not your submission contains all raw data required to replicate the results of your study. Authors must share the “minimal data set” for their submission. PLOS defines the minimal data set to consist of the data required to replicate all study findings reported in the article, as well as related metadata and methods (<https://journals.plos.org/plosone/s/data-availability#loc-minimal-data-set-definition>).

For example, authors should submit the following data:

- The values behind the means, standard deviations and other measures reported;

Sensitivity and specificity values are based on multiple sources. Raw data from these sources and information about the calculation of means are added to the supplementary files, please see Appendix B, tables 1-4.

- The values used to build graphs;
- The points extracted from images for analysis.

Authors do not need to submit their entire data set if only a portion of the data was used in the reported study.

If your submission does not contain these data, please either upload them as Supporting Information files or deposit them to a stable, public repository and provide us with the relevant URLs, DOIs, or accession numbers. For a list of recommended repositories, please see <https://journals.plos.org/plosone/s/recommended-repositories>.

The authors hereby confirm that all data needed to replicate this study is submitted to PlosOne.

If there are ethical or legal restrictions on sharing a de-identified data set, please explain them in detail (e.g., data contain potentially sensitive information, data are owned by a third-party organization, etc.) and who has imposed them (e.g., an ethics committee). Please also provide contact information for a data access committee, ethics committee, or other institutional body to which data requests may be sent. If data are owned by a third party, please indicate how others may request data access.

This is not applicable to our study.

5. Please upload a new copy of Figure 1 as the detail is not clear. Please follow the link for more information:

https://blogs.plos.org/plos/2019/06/looking-good-tips-for-creating-your-plos-figures-graphics/
<https://blogs.plos.org/plos/2019/06/looking-good-tips-for-creating-your-plos-figures-graphics/>

Please see the file Figure 1_revised.png. The quality of the image should be better now, our apologies for the inconvenience.

**Additional Editor Comments:**

This study provide additional reference for the early-stage assessment of FFR, which is an interesting topic. However it need further improvement. Please carefully check the reviewers' comments.
In addition, the following points should be considered.
1. There are some format errors (.Error! Bookmark not defined) in the tables.

Thank you for this remark and for reviewing our manuscript. However, the version of Table 1 as shared with PlosOne does not show any format errors. Please see the screenshots below:

2. In Table 1, I recommend to unify the digits of numbers.

We agree with the reviewer that it looks better when digits are unified. We unified the digits in Table 1 as much as possible, based on available data.

Please see Table 1 on pages 8-9.

3. FFRct is calculated based on CT images. Often the CT scan is recommended to the patients with symptoms. Therefore, regarding the one-year probability of MACE, the low value for FFTct need more discussion.

Thank you for this comment. In the introduction it is explained that FFRct adds a mathematical simulation to the CT scan, and thereby increases the accuracy of the test.

Please see page 5, lines 70-75:

*To prevent unnecessary additional or invasive diagnostic procedures, measuring FFR from the non-invasive CCTA (FFRct) has been developed. With data from the CCTA scan, FFRct assesses the impact of stenosis in the coronaries using imaging data and mathematical simulations of the blood flow. Because of its accuracy, it could prevent unnecessary and costly treatments, including revascularizations, and diagnostic procedures without adding the burden and adverse events of an invasive technique [7-9].*

Risk of MACE after an FFRct scan are lower than a CT scan due to the increased diagnostic accuracy of FFRct. To clarify this, it is added to the discussion section.

Please see page 15, lines 292-294:

*Since FFRct adds a mathematical simulation to the traditional CCTA, thereby increasing the accuracy of the diagnostic test, it is always expected to lower the amount of MACE.*

4. For the included literature, the inclusion and exclusion criteria need further clarification. The authors can consider a literature screening flowchart.

Thank you for this comment. The inclusion and exclusion criteria used in the literature review are now added to the methods and results sections.

Please see page 6, lines 99-106:

*To collect data on costs and effects a literature search was performed in Pubmed using the following search terms: (((coronary artery disease[MeSH Terms]) AND (cardiac diagnostic method[MeSH Terms])) AND ((“2015/01/01”[Date – Completion] : “3000”[Date – Completion]))) AND ((coronary angiography[MeSH Terms]) OR (fractional flow reserve, myocardial[MeSH Terms]) OR (computed tomography[MeSH Terms])). Articles were excluded if they were published before 2015, if they did focus on other coronary malignancies than stable chest pain, and if they were not designed as a meta-analysis, systematic review or randomized controlled trial.*

And page 12, lines 203-207:

***Literature review***

*The literature review resulted in 197 results. After title and abstract screening, 162 articles were excluded. After full text review, twelve articles were selected for inclusion, see Figure 2.*

*Figure 2. Flow chart of literature review*

[Note: HTML markup is below. Please do not edit.]

Reviewers' comments:

Reviewer's Responses to Questions

**Comments to the Author**

1. Is the manuscript technically sound, and do the data support the conclusions?

The manuscript must describe a technically sound piece of scientific research with data that supports the conclusions. Experiments must have been conducted rigorously, with appropriate controls, replication, and sample sizes. The conclusions must be drawn appropriately based on the data presented.

Reviewer #1: Yes

Reviewer #2: Yes

2. Has the statistical analysis been performed appropriately and rigorously?

Reviewer #1: Yes

Reviewer #2: Yes

3. Have the authors made all data underlying the findings in their manuscript fully available?

The [PLOS Data policy](http://www.plosone.org/static/policies.action#sharing) requires authors to make all data underlying the findings described in their manuscript fully available without restriction, with rare exception (please refer to the Data Availability Statement in the manuscript PDF file). The data should be provided as part of the manuscript or its supporting information, or deposited to a public repository. For example, in addition to summary statistics, the data points behind means, medians and variance measures should be available. If there are restrictions on publicly sharing data—e.g. participant privacy or use of data from a third party—those must be specified.

Reviewer #1: Yes

Reviewer #2: Yes

4. Is the manuscript presented in an intelligible fashion and written in standard English?

PLOS ONE does not copyedit accepted manuscripts, so the language in submitted articles must be clear, correct, and unambiguous. Any typographical or grammatical errors should be corrected at revision, so please note any specific errors here.

Reviewer #1: Yes

Reviewer #2: Yes

5. Review Comments to the Author

Please use the space provided to explain your answers to the questions above. You may also include additional comments for the author, including concerns about dual publication, research ethics, or publication ethics. (Please upload your review as an attachment if it exceeds 20,000 characters)

Reviewer #1: This paper assessed the difference in healthcare costs and effects of fractional flow reserve derived from coronary computed tomography (FFRct) compared to standard diagnostics in patients with stable chest pain in the Netherlands. It is an excellent work. However, I have some suggestions.
1. Please provide more details about the decision-tree model.

The model is described in the methods section and illustrated in Figure 1.

Please see pages 6-7, lines 97-128:

*Model overview*

*A decision-tree model was developed that integrated data from published literature and information about unit costs to compare costs and clinical outcomes of FFRct compared to standard diagnostics**. To collect data on costs and effects a literature search was performed in Pubmed using the following search terms: (((coronary artery disease[MeSH Terms]) AND (cardiac diagnostic method[MeSH Terms])) AND (("2015/01/01"[Date - Completion] : "3000"[Date - Completion]))) AND ((coronary angiography[MeSH Terms]) OR (fractional flow reserve, myocardial[MeSH Terms]) OR (computed tomography[MeSH Terms])). Articles were excluded if they were published before 2015, if they did focus on other coronary malignancies than stable chest pain, and if they were not a meta-analysis, systematic review or randomized controlled trial. The model simulates different diagnostic trajectories, including non-invasive anatomical imaging (i.e., CCTA), non-invasive functional imaging (i.e., magnetic resonance imaging (MRI)), and ICA with visual assessment and/or combined with a FFR measurement over a period of one year. The FFRct diagnostic strategy added the computational FFR analysis to the initial CCTA for all patients. Model outcomes included total costs from the hospital perspective, probability of correct diagnoses,* *and risk of major adverse cardiovascular events (MACE), defined as all-cause mortality, myocardial infarction (MI), and unplanned hospitalization for chest pain leading to urgent revascularization [8]. Outcomes were not discounted. The model was constructed in TreeAge Pro (version 2020; Williamstown, MA: TreeAge Software, Inc.).*

*Patients with stable chest pain and an intermediate pre-test probability of obstructive CAD enter the model and received a CCTA (with or without FFR analysis depending on the diagnostic arm) (****Figure 1****). If the test findings were inconclusive, meaning the test provided insufficient information about the effect of the lesion on the blood flow (i.e., no positive or negative test result), patients will undergo non-invasive functional imaging (i.e., MRI). If those findings are also inconclusive, patients will undergo an ICA with visual assessment. Finally, when those test findings were inconclusive, patients received ICA with an FFR measurement, in line with the transmural agreements for patients with non-acute chest pain of the Dutch Association of Cardiologist [6, 11]. Patients who received a positive test result (either true or false) after a test were assumed to be treated with medications or a percutaneous coronary intervention (PCI) or coronary artery bypass grafting (CABG). Patients who received a negative test result (either true or false) receive no further treatment. Patients with a conclusive test result (positive or negative) were at risk of MACE.*

It is unclear to us what more details about the decision tree-model need to be described.

2. What specific guiding value can this research provide to the clinic? Please discuss some suggestions for FFRCT technology in the future.

Thank you for this suggestion. We elaborated on the possible value this study could provide to a clinic in the discussion section.

Please see page 17, lines 324-330:

*For hospitals diagnosing patients with stable chest pain in The Netherlands, the results of this study indicate that the implementation of FFRct guided diagnostics would decrease the costs per patient, the amount of adverse events and increase the probability of correct diagnoses.* *For hospitals in the Netherlands considering to implement FFRct, this study shows promising results. Moreover, these study findings may contribute to the decision making process by the Dutch National Health Care Institute about reimbursement of FFRct as part of the standard health care package [31].*

3. Figures are not clear, please provide high resolution pictures.

Thank you for noticing. We reviewed and re-uploaded all Figures and trust they are now in sufficient resolution.

Reviewer #2: Good preliminary work of comparing the cost of HTA of FFRCTA against traditional diagnostic workflow of stable angina. Perhaps can include in discussion cost implications of this technology in resource limited environments/ expansion of this strategy to Asia Pacific (APAC) countries.

Thank you for reviewing our manuscript and your kind words. Since our study focussed on the situation in The Netherlands, we currently do not have enough information to make statements about other situations. Nonetheless, we added a sentence to the discussion section to emphasize that different situations require a new analysis to be able to assess the potential value of FFRct.

Please see page 17, lines 330-332:

*The authors would like to emphasize that this study focused on the healthcare setting in The Netherlands, therefore other settings require an analysis tailored to those specific settings.*

6. PLOS authors have the option to publish the peer review history of their article ([what does this mean?](https://journals.plos.org/plosone/s/editorial-and-peer-review-process#loc-peer-review-history)). If published, this will include your full peer review and any attached files.


If you choose “no”, your identity will remain anonymous but your review may still be made public.


**Do you want your identity to be public for this peer review?** For information about this choice, including consent withdrawal, please see our [Privacy Policy](https://www.plos.org/privacy-policy).

Reviewer #1: No

Reviewer #2: **Yes:** Han Bing Chow
